# The Druggable Pocketome of *Corynebacterium diphtheriae*: A New Approach for *in silico* Putative Druggable Targets

**DOI:** 10.3389/fgene.2018.00044

**Published:** 2018-02-13

**Authors:** Syed S. Hassan, Syed B. Jamal, Leandro G. Radusky, Sandeep Tiwari, Asad Ullah, Javed Ali, Behramand, Paulo V. S. D. de Carvalho, Rida Shams, Sabir Khan, Henrique C. P. Figueiredo, Debmalya Barh, Preetam Ghosh, Artur Silva, Jan Baumbach, Richard Röttger, Adrián G. Turjanski, Vasco A. C. Azevedo

**Affiliations:** ^1^Department of Chemistry, Islamia College University Peshawar, Peshawar, Pakistan; ^2^PG Program in Bioinformatics, Institute of Biological Sciences, Federal University of Minas Gerais, Belo Horizonte, Brazil; ^3^Departamento de Química Biológica, Facultad de Ciencias Exactas y Naturales, Universidad de Buenos Aires, Buenos Aires, Argentina; ^4^Department of Chemistry, Kohat University of Science and Technology, Kohat, Pakistan; ^5^Department of Analytical Chemistry, Institute of Chemistry, São Paulo State University, São Paulo, Brazil; ^6^AQUACEN, National Reference Laboratory for Aquatic Animal Diseases, Ministry of Fisheries and Aquaculture, Federal University of Minas Gerais, Belo Horizonte, Brazil; ^7^Centre for Genomics and Applied Gene Technology, Institute of Integrative Omics and Applied Biotechnology, Purba Medinipur, India; ^8^Department of Computer Science, Virginia Commonwealth University, Richmond, VA, United States; ^9^Institute of Biological Sciences, Federal University of Pará, Belém, Brazil; ^10^Department of Mathematics and Computer Science, University of Southern Denmark, Odense, Denmark; ^11^INQUIMAE/UBA-CONICET, Facultad de Ciencias Exactas y Naturales, Universidad de Buenos Aires, Buenos Aires, Argentina

**Keywords:** *Corynebacterium diphtheria*, pocketome, druggable genome, structural proteomics, putative therapeutic targets, highly druggable (HD), global druggable (GD)

## Abstract

Diphtheria is an acute and highly infectious disease, previously regarded as endemic in nature but vaccine-preventable, is caused by *Corynebacterium diphtheriae* (Cd). In this work, we used an *in silico* approach along the 13 complete genome sequences of *C. diphtheriae* followed by a computational assessment of structural information of the binding sites to characterize the “pocketome druggability.” To this end, we first computed the “modelome” (3D structures of a complete genome) of a randomly selected reference strain Cd NCTC13129; that had 13,763 open reading frames (ORFs) and resulted in 1,253 (∼9%) structure models. The amino acid sequences of these modeled structures were compared with the remaining 12 genomes and consequently, 438 conserved protein sequences were obtained. The RCSB-PDB database was consulted to check the template structures for these conserved proteins and as a result, 401 adequate 3D models were obtained. We subsequently predicted the protein pockets for the obtained set of models and kept only the conserved pockets that had highly druggable (HD) values (137 across all strains). Later, an off-target host homology analyses was performed considering the human proteome using NCBI database. Furthermore, the gene essentiality analysis was carried out that gave a final set of 10-conserved targets possessing highly druggable protein pockets. To check the target identification robustness of the pipeline used in this work, we crosschecked the final target list with another in-house target identification approach for *C. diphtheriae* thereby obtaining three common targets, these were; hisE-phosphoribosyl-ATP pyrophosphatase, glpX-fructose 1,6-bisphosphatase II, and rpsH-30S ribosomal protein S8. Our predicted results suggest that the *in silico* approach used could potentially aid in experimental polypharmacological target determination in *C. diphtheriae* and other pathogens, thereby, might complement the existing and new drug-discovery pipelines.

## Introduction

*Corynebacterium diphtheriae* belong to the class Actinomycetales and is a Gram-positive bacterium, a non-spore forming, non-motile and facultative anaerobe with pleomorphic cell shape and high GC content (∼53%) relative to the *Firmicutes* (Cerdeno-Tarraga et al., 2003; Trost et al., 2012). This bacterium is the causative agent of diphtheria, a severe human respiratory manifestation characterized by bacterial adhesion to host pharyngeal cell (pharyngitis and pseudomembranous inflammation). The pathogen target one or both tonsils that further disseminate at a later stage thereby resulting in complete airway obstruction and death (Hadfield et al., 2000). The cornerstone in diphtheria therapy involves the hyper immune antiserum-antitoxin produced in equines that neutralizes the *C. diphtheriae* toxin while among antibiotics are the broad-spectrum penicillin or erythromycin. However, recent emergence of numerous drug-resistant strains subsequently decreases the efficacy of current therapeutics (Barraud et al., 2011). Importantly, the World Health Organization recommends widespread DTPs immunization against toxigenic diphtheria strains as the only effective approach to counteract the infection. Although, a slight change in mortality has been observed since the availability and administration of antibiotics, specifically penicillin or erythromycin (Adler et al., 2013). *C. diphtheriae* has four biotypes: gravis, mitis, intermedius and belfanti that are non-sporulating, unencapsulated, non-motile and pleomorphic bacilli. They are subsequently classified on the basis of differences in colonial morphology, haemolytic potential, fermentation reactions and severity of the resulting disease (Gerald et al., 2009; Brooks et al., 2010). The infant mortality rate in an epidemic that resulted in thousands of casualties dropped gradually in countries where living standards were improved and immunization programs were introduced. Nevertheless, it still remains a significant pathogen around the globe (Hodes, 1979). The ‘strangling angel’ effects on children that scaled from wing-shaped disarticulation and pseudo-membranes formation in the oropharynx, triggered acute obstruction of airways and resulted in mortalities (Hodes, 1979; Hart et al., 1996; Jamal et al., 2017b). A plethora of cases were recently reported and still expected from both non-lethal and lethal diphtheria in different parts of the world due to significant population displacements via immigration. An adequate supervision necessitates quick measures to discover additional diphtheria antitoxin, antibiotic and therapeutic treatment (Pizza et al., 2000).

The emerging concepts of polypharmacology, differential genome analyses, and reverse vaccinology, comparative and subtractive microbial genomics have largely contributed by establishing complementary traditions for fast identification of novel targets in post-genomic era (Perumal et al., 2007; Barh et al., 2013). Comparative homology modeling (Baumbach, 2010; Rottger et al., 2013) has widely been used in expanding the structural space of pathogens (Chong et al., 2006; Asif et al., 2009).

These practices are being used for the identification of conserved targets in a several human and animal pathogens like *C. tuberculosis* (Hassan et al., 2014; Radusky et al., 2015), *Mycobacterium. tuberculosis* (Asif et al., 2009), *Burkholderia pseudomallei* (Chong et al., 2006), *Neisseria gonorrhoea* (Barh and Kumar, 2009), *Helicobacter pylori* (Dutta et al., 2006), *Pseudomonas aeruginosa* (Sakharkar et al., 2004; Perumal et al., 2007), and *Salmonella typhi* (Rathi et al., 2009).

In this work, a combination of *in silico* tools was primarily used to predict the core proteome of *C. diphtheriae* species to associate genomic information based on the 3D structures. The predicted proteomes were modeled (pan-modelome) using a methodology adapted by Hassan et al. (2014). From a structural point of view, druggability is the probability of small drug-like molecules binding to a given target protein with high affinity (<1 μM). We report for the first time the structural druggability assessment for multi-strain *C. diphtheriae* proteomes using a pan-druggability prediction pipeline based on the open source pocket detection code “fpocket”. The method integrates several physicochemical descriptors to estimate the pocket druggability on a genomic scale with suitable features that enable binding of a drug-like compound (Kinnings et al., 2010).

## Materials and Methods

### Initial Dataset Construction

All ORFs (Open Reading Frames) of the 13 completely sequenced genomes of *C. diphtheriae* were obtained from the NCBI database^1^. **Table 1** shows the statistical data of all strains used in this study where the strain NCTC13129 has 2,272 reported ORFs and was randomly selected as a reference genome for modelome prediction and further analyses.

**Table 1:** Summary of *Corynebacterium diphtheriae* strains used in this study and their respective modeling statistics for druggability analyses.

Strain	Biovar	Location	NCBI accession	Genome size (Mb)	GC%	Proteins	Models – G2
31A	N/A	Brazil	NC_016799.1	2.53535	53.60	2380	1283
241	N/A	Brazil	NC_016782.1	2.42655	53.40	2245	1235
BH8	N/A	Brazil	NC_016800.1	2.48552	53.60	2361	1269
C7	N/A	United States	NC_016801.1	2.49919	53.50	2337	1278
CDCE8392	Mitis	United States	NC_016785.1	2.43333	53.60	2249	1253
HC01	Mitis	Brazil	NC_016786.1	2.42715	53.40	2247	1236
HC02	Mitis	Brazil	NC_016802.1	2.46861	53.70	2230	1254
HC03	Mitis	Brazil	NC_016787.1	2.47836	53.50	2262	1260
HC04	Gravis	Brazil	NC_016788.1	2.48433	53.50	2275	1260
INCA402	Belfanti	Brazil	NC_016783.1	2.44907	53.70	2214	1282
PW8	N/A	United States	NC_016789.1	2.53068	53.50	2414	1272
VA01	Gravis	Brazil	NC_016790.1	2.39544	53.40	2191	1239
NCTC13129	Gravis	United Kingdom	NC_002935.2	2.48863	53.50	2272	1253

### General Concept: Modelome Prediction

**FIGURE 1:**
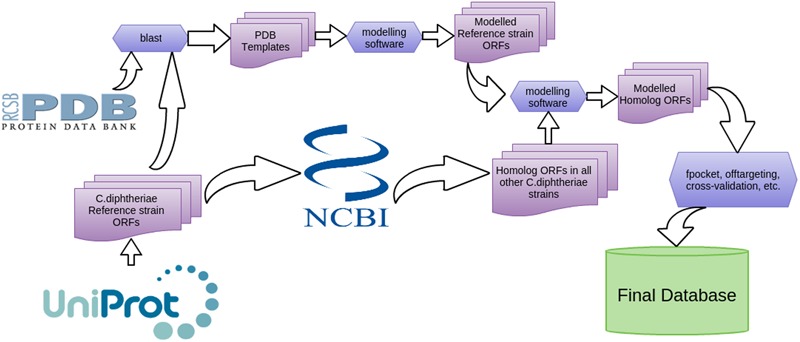
Overview of pipeline components for data analysis.

The binding affinity of small drug-like molecules to the active site of putative biological targets (druggable protein cavities) formulated a basis for this work, a slightly modified protocol of Radusky et al. (2015) (**Figure 1**). All genome ORF sequences of the 13 *C. diphtheriae* strains were subjected to the MHOLline workflow^2^ in.faa file format for 3D structure prediction. MHOLline utilizes multi fasta files of amino acids as an input data and then uses HMMTOP, BLAST, BATS, Modeller and Procheck programs for the detailed analyses. The program HMMTOP detects transmembrane regions. The BLAST algorithm is used to identify template structure by performing a random search against the Protein Data Bank. BATS (Blast Automatic Targeting for Structures) carry out the refinement in the template search; it is a key step for the model construction. BATS refinement identifies sequences that make the modeling possible by selecting a template from BLAST output file using their BATS scores, expectation values, identity and sequence similarity as criteria as well as considering the number of gaps and the alignment coverage. BATS select the best template for 3D model generation and perform automated alignment used by the MODELLER program. Furthermore, it gathers all the BLAST output files into four distinctive groups, i.e., G0, G1, G2, and G3, according to the following criteria; G0 = Not aligned sequence, G1 = *E*-value > 10e^-5^ or Identity < 15%, G2 = *E*-value ≤ 10e^-5^ and Identity ≥ 25% AND LVI ≤ 0.7, G3 = *E*-value ≤ 10e^-5^ and Identity ≤ 15% and <25% OR LVI > 0.7, Where LVI is the Length Variation Index, a MHOLline concept of coverage (LVI ≤ 0.1 is equivalent to a coverage ≥ 90%). Once the template is selected based on BATS results, MODELLER program is used for the generation of 3D protein model. There is no significant correlation statistically, between the number of templates used during model building and the overall quality of a model. In the next step, another MHOLline tool called FILTERS, categorizes the BATS selected sequences (G2) into distinct quality model subgroups, based on identity and LVI value. The subgroups ranges Very High to Very Low. To evaluate the overall quality and accuracy of the model, Ramachandran plot is obtained which explained the stereochemical quality of the model. Precisely, the MHOLine generates an aggregate structural information for all the submitted sequences in the fasta format, Ramachandran plot and other properties like structural quality and enzymatic functions are also determined. Further details can be obtained by visiting MHOLline homepage (Hassan et al., 2014; Webb and Sali, 2016; Jamal et al., 2017a). For all modeled structures, structural properties were figured as: (i) the Druggability Score (DS) for each pocket and (ii) the active site residues (if available) according to the template structures available at the protein databank RCSB-PDB^3^ (Berman et al., 2003).

### 3D Protein Models in Non-reference Strains: Orthologs Identification

We used an applied bioinformatics procedure to find the conserved putative druggable targets across all the 13 *C. diphtheriae* strains at genome-scale by first predicting their 3D models. The ensemble methodology essentially is a filter of thousands of candidate genes to yield high-confidence 3D structural models from orthologous proteins in *C. diphtheriae* species. As aforementioned, the MHOLline resulted in 1,253 predicted structures for the randomly selected reference strain NCTC13129 that were later used as template structures for modeling the 3D structures in non-reference strains as well. Further, the BLASTp program was installed in a local machine and used to check if the ORFs of the reference proteome have orthologs in the remaining 12 strains using the following parameters; identity ≥ 85%, coverage > 80%. The protein sequences showing high identity values (> = 85%) for each reference and non-reference strain were considered as conserved and the modeled structures of reference strain were used again as templates to predict the 3D models for the aforementioned 12 non-reference strains. The core modelome was compared and evaluated for the quality of the obtained 3D structures. A reliable model has a probability of correct fold larger than 95% and coverage of over 50% with the template structure. For each sequence in the reference and non-reference strain that gave an identity hit of >85%, a mutation methodology was applied on each amino acid substitution using the MODELLER program. These models were then used to compute the druggability variation for the 13 strains of the *C. diphtheriae* species.

### Evaluation of Structural Druggability

The protein structural druggability of each predicted 3D model was evaluated by determining the ability of putative pockets to bind drug-like molecule/s, using the fpocket (Finn et al., 2016) and the recently developed DrugScore (DS) programs (Velec et al., 2005). The later methodology is based on the Voronoi tessellation algorithm that identify and characterize pockets and compute suitable physicochemical descriptors such as polar and apolar surface area, hydrophobic-hydrophobic density and polarity score. In conjunction they yield a druggability value that ranges between 0 (non-druggable, ND) and 1 (highly druggable, HD). We categorized the druggability scores for all predicted 3D structures into four sets: non-druggable (ND; DS ≤ 0.2), poorly druggable (PD; DS ≥ 0.2 and ≤0.5), druggable (D; DS ≥ 0.5 and ≤0.7), and highly druggable (HD; DS ≥ 0.7) protein pockets, respectively. This distribution is in accordance to our previous work where the druggability score was computed for all pockets present in all unique proteins in the Protein Data Bank that were experimentally crystallized in complex with a drug-like compound/s (Radusky et al., 2014).

### Identification of Active Site Residues

In order to identify the amino acid residues in the active site of the predicted druggable protein pocket/s, information were retrieved from the CSA database (Catalytic Site Atlas) (Furnham et al., 2014) and Pfam position site (Finn et al., 2016), respectively. A list of PDB_IDs was generated linked to a number of residues constituting the corresponding protein active sites. To map the active site residues to as many *C. diphtheriae* proteins as possible, each PDB_ID was used as a template in CSA and assigned to the modeled ORFs.

### Host Homology, Essentiality, and Core-Modelomics of the Selected Targets

For off target prediction, the pool of global druggable (GD) proteins was piped into NCBI-BLASTp using default parameters (identity = 0% and/or no hit) against the human proteome to identify non-host homologs. Moreover, from the filtered list of 10 highly druggable non-host homologous target proteins, an approach based on subtractive genomics was implemented and applied to the GD targets that were essential to bacteria (Barh et al., 2011). Briefly, the set of target proteins of *C. diphtheriae* was submitted to the Database of Essential Genes (DEG, which contains experimentally validated essential genes from bacteria, archaea and eukaryotes) for homology analyses (Zhang et al., 2004). Again, we used BLASTp with *E-*value cut-off of 1e^-05^ a *bit score* ≥ 100 and *identity* ≥ 35% (Barh et al., 2011). The final list of putative targets based on criteria described earlier, contained 10 essential and non-host homologous target proteins. The obtained list was further subjected to ProtParam^4^ for molecular weight determination, biochemical pathway analysis to KEGG (Kyoto Encyclopedia of Genes and Genomes) (Kanehisa and Goto, 2000) using network enrichment (Alcaraz et al., 2012), virulence using PAIDB (Pathogenicity Island Database) (Yoon et al., 2007), functionality using UniProt (Universal Protein Resource) (Magrane and UniProt, 2011), and cellular localization using CELLO (subCELlular LOcalization predictor) (Yu et al., 2004). In addition, we merged the final set of 10 selected non-host homologous, essential and global druggable proteins with results obtained through experiments locally performed in our laboratories (Jamal et al., unpublished data) resulting in three common targets, which we selected as candidates.

### Protein–Protein Interaction Network

In biological systems, proteins work in a homogenous environment rather than individual, hence it is important to study protein–protein interactions (PPIs) for *C. diphtheriae* metabolism. The identified drug targets were evaluated to study their potential biological, functional and metabolic roles for proteomic interactions. The selected drug targets were used to develop intra-species protein–protein interactome using STRING (Search Tool for the Retrieval of Interacting Genes/Proteins) database (Szklarczyk et al., 2015). STRING is an online network analyses tool that provides essential information regarding interactions of the desired proteins.

## Results and Discussion

### Prediction of Structural Homology Based Models

The complete modelome of the reference strain NCTC13129 was computed; consisting of 13,763 ORF, with 1,253 (∼9%) resulting models. Taking the original models of reference strain NCTC13129 as templates, we then generated 438 conserved models in the 12 remaining strains using the MODELLER software (Sali and Blundell, 1993). Afterward, the target-template alignments have been computed using a BLAST *E-*value cut-off of 10^-6^ in order to build the model structures using the MODELLER software (Sali and Blundell, 1993; Webb and Sali, 2016). For each target-template alignment, ten different target models were built, and their quality measures have been assessed using GA341 (Melo and Feytmans, 1998; Melo and Sali, 2007) and QMEAN (Benkert et al., 2009), keeping models with GA341 reliability scores ≥ 0.7 (Melo et al., 2002), leading to a final set of 401 protein models. All these proteins are tabulated in **Supplementary Table S1**.

### Pocketome Druggability and Active Site Residues of *C. diphtheriae*

The list of 401 targets protein drastically reduced to 137 after druggability analyses using the aforementioned fpocket and the recently developed DS programs. A summary of only highly druggable (HD) targets with drug score remained ≥ 0.7 were considered as global druggable. The calculated structural druggability scores are given in **Supplementary Table S2**.

**FIGURE 2:**
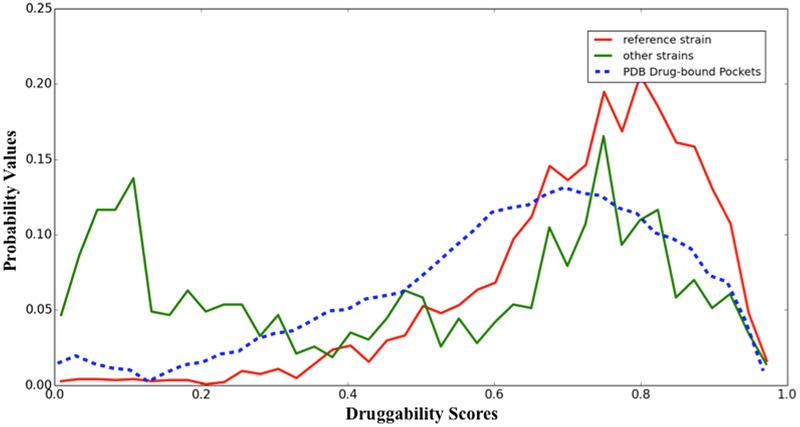
Histogram of druggability score along the *Corynebacterium diphtheriae* reference/other strains. Representation of all ligand-bound structures in the PDB (blue pointed line), all modeled structures of *C. diphtheriae* reference strain (red line, 1,253 models), and all modeled structures of the *C. diphtheriae* non-reference genome strains (green line, 401 core proteins in 12 non-reference strains).

In **Figure 2**, a comparison of calculated druggability score distribution across all structures of *C. diphtheriae* reference and other strains is shown. Although the distribution has a small shift to higher values, we used the same bounds to define the sets of druggable proteins (**Figure 2**). A protein target, which remained druggable in all strains, was classified as Globally Druggable (GD).

### Non-host Homology, Essentiality and Core-Modelomics Analyses

As aforementioned, the list of 137 global druggable proteins (**Supplementary Table S2**) was computed to the corresponding human host proteome that resulted in the identification of a very small set of only 10 non-host homologous proteins; remaining 127 as host-homologous. The non-host homologous targets were selected following a very stringent criterion, i.e., no identity, no hits. This list of final 10 essential and non-host homologous targets in *C. diphtheriae* is given in **Table 2**. We further report the involvement of these putative targets in metabolic pathways, biological processes, cellular localization, molecular weights and most importantly their potential role as virulence factors. Out of 10 targets, 7 targets were found as pathogen virulence factors using the PAIDB database based on homology method. Further, we predicted the subcellular localization of these final target proteins using support vector machines, a methodology that is based on n-peptide composition of proteins, adapted in a related work by Yu et al. (2004), we obtained three high-confidential candidates, which are; hisE, glpX, and rpsH. Interestingly, these three high-confidential candidates were identified as essential and non-host homologous targets in our previous work by Jamal et al. (2017a). These proteins were subjected to molecular docking analysis against four different ligand libraries and a set of some potent molecules were suggested for active inhibition of these proteins (Jamal et al., 2017a).

**Table 2:** List of global druggable, non-host homologous and essential putative targets, their functional annotation together with other information.

S. No	Gene/protein codes	Official full name	Mol. Wt^a^ (KDa)	Functions^b^	Cell locality^c^	Pathways^d^	Virulence^e^
1	NP_939496.1 coaD	Phosphopantetheine adenylyltransferase	17.305	**MF:** ATP binding, pantetheine-phosphate adenylyltransferase activity. **BP:** coenzyme A biosynthetic process.	Cytoplasm	Pantothenate and CoA biosynthesis, metabolic pathways	Yes
**2**	NP_939612.1 **hisE**	Phosphoribosyl-ATP pyrophosphatase	9.877	**MF:** RNA binding, phosphoribosyl-ATP diphosphatase activity. **BP:** histidine biosynthetic process	Cytoplasm	Biosynthesis of amino acids	Yes
3	NP_938944.1 DIP0568	Alanine racemase	41.235	**MF:** Catalyzes the interconversion of L-alanine and D-alanine. May also act on other amino acids. **BP:** D-alanine biosynthetic process	Cytoplasm	D-Alanine metabolism, Metabolic pathways, Vancomycin resistance	Yes
4	NP_939875.1 rimM	16S rRNA-processing protein RimM	18.067	**MF:** ribosome binding. **BP:** ribosomal small subunit biogenesis, rRNA processing	Cytoplasm	Ribosome biogenesis	No
**5**	NP_939302.1 **glpX**	Fructose 1,6-bisphosphatase II	35.589	**MF:** fructose 1,6-bisphosphate 1-phosphatase activity, metal ion binding. **BP:** gluconeogenesis, glycerol metabolic process	Cytoplasm	Carbohydrate metabolism	No
6	NP_940458.1 dcd	Deoxycytidine triphosphate deaminase	20.494	**MF:** dCTP deaminase activity. **BP:** dUMP biosynthetic process, dUTP biosynthetic process, pyrimidine ribonucleotide biosynthetic process	Cytoplasm	Metabolism	Yes
**7**	NP_938900.1 **rpsH**	30S ribosomal protein S8	14.292	**MF:** rRNA binding, structural constituent of ribosome. **BP:** translation	Extracellular/ Cytoplasm	Unknown	No
8	NP_938817.1 rplJ	50S ribosomal protein L10	17.946	**MF:** large ribosomal subunit rRNA binding, structural constituent of ribosome. **BP:** ribosome biogenesis, translation	Cytoplasm	Genetic information processing, translation	Yes
9	NP_940678.1 rsmG	16S rRNA methyltransferase GidB	24.425	**MF:** rRNA (guanine-N7-)-methyltransferase activity. **BP:** rRNA processing	Cytoplasm	Methyltransferases	Yes
10	NP_938439.1 thiE	Thiamine-phosphate synthase	23.441	**MF:** magnesium ion binding, thiamine-phosphate diphosphorylase activity. **BP:** thiamine biosynthetic process, thiamine diphosphate biosynthetic process	Cytoplasm	Thiamine metabolism, metabolic pathways	Yes

*The underlined are the three high-confidence targets (see text).*

*^*a*^Molecular weight via ProtParam tool (http://web.expasy.org/protparam/).*

*^*b*^Molecular function (MF) and biological process (BP) for each target protein via UniProt.*

*^*c*^Cellular localization of pathogen targets via CELLO.*

*^*d*^KEGG for finding the role of these targets in different cellular pathways.*

*^*e*^PAIDB for analyzing if the putative targets are involved in the pathogen’s virulence.*

#### hisE (Phosphoribosyl-ATP Pyrophosphatase)

hisE is the second enzyme in histidine-biosynthetic pathway hydrolysing irreversibly phosphoribosyl-ATP to phosphoribosyl-AMP and pyrophosphate. The protein is encoded by the *hisE* gene, fused to *hisI* in many bacteria, fungi and plants but is present as a separate gene in some bacteria and archaea. Since it is seen in *in vitro* experiments that *hisE* is essential for microorganism growth, we assume it a potential drug target in *C. diphtheriae.* It is also reported as a drug candidate for tuberculosis (Javid-Majd et al., 2008).

#### glpX (Fructose 1,6-Bisphosphatase II)

It is one of the main enzyme for gluconeogenesis that catalyses the hydrolysis of fructose 1,6-bisphosphate to form fructose 6-phosphate and orthophosphate. In glycolysis, phosphofructokinase catalysis the reverse reaction, and the product, fructose 6-phosphate, are important precursors in various biosynthetic pathways (Horecker et al., 1975). Gluconeogenesis is an important metabolic pathway in all organisms and plays a key role by allowing the cells to synthesize glucose from non-carbohydrate precursors, such as glycerol, organic acids and amino acids. FBPases are members of lithium sensitive phosphatases a large superfamily which includes three families of inositol phosphatases and FBPases (phosphoesterase clan CL0171, AA sequences 3167 from Pfam data base). They are already reported as target for the treatment of non-insulin dependent diabetes and development of new drugs (Wright et al., 2002; Sassetti and Rubin, 2003).

#### rpsH (30S Ribosomal Protein S8)

The protein rpsH is one of the key RNA-binding protein having a central position within the small ribosomal subunit. It interacts widely with 16S rRNA and is fundamental for the correct folding of the central domain of the ribosomal rRNA. Furthermore, this protein regulates the synthesis of various other ribosomal proteins by binding to mRNA. In the two RNA molecules, it binds exactly to very similar sites. rpsH has a medium size and recently it has been discovered that rpsH play vital role as a significant primary RNA-binding protein in the 30S subunit. Mutations in S8 within the protein are shown to result in defective ribosomal assembly. The S8-binding site within 16S rRNA in *Escherichia coli*, has been investigated independently by a number of techniques including protein crosslinking, nuclease protection, hydroxyl-radical foot printing, RNA–RNA modification and chemical probing. The 30S ribosomal protein S8 is also one of the principal regulatory elements that control ribosomal protein synthesis by the translational feedback inhibition mechanism discovered by Yates et al. (1980). It regulates the expression of spc operon that encodes the 10 ribosomal proteins L5, L6, L14, L15, L18, L24, L30, S5, S8, and S14, respectively (Davies et al., 1996).

### Protein–Protein Interaction Network for Proposed Targets

Protein–protein interaction of target proteins with each other have been constructed showing two proteins, rplJ (VN94_02905/50S ribosomal protein L10) and rpsH (30S ribosomal protein S8) to be interacting directly based on highest confidence score 0.9. The confidence score is the approximate probability that a predicted link exists between two enzymes in the same metabolic map in the KEGG database. The evidences for this interaction are gene fusion, co-occurrence, co-expression, experimental and databases (**Supplementary Figure S1**).

## Conclusion

We performed a comprehensive *in silico* study of the druggability scores on all sequenced genomes of *C. diphtheria* resulting in a list of intra-strain highly druggable pockets of 10 ORFs non-homologous in human hosts. Previously, we have implemented a similar approach using other bioinformatics tools for the identification of putative therapeutic targets in *C. diphtheriae* that relied primarily on the modelome construction followed by filtering the obtained data for conserved targets (Jamal et al., 2017a). In that work, a final set of eight essential and non-host homologs targets were subjected to virtual screening using different compound libraries but lacked a detailed overview of the druggable protein pockets of the selected targets. Here, we further extrapolated our work to the druggable pocketome at species level and then at the end compared our final data set obtained in this work with the aforementioned published data. The comparison showed that any of the two approaches for putative targets identification in pathogenic microorganisms might provide an easy-to-handle protocol in future drug discovery projects. Our pipeline is expandable and can be applied to other bacterial species as well. In the future, we will work on Cytoscape plugins to allow for mapping essential druggable non-homologous genes to biological networks interactively for follow-up systems biology investigation (Baumbach and Apeltsin, 2008). We believe that our approach has the potential to aid in designing drugs and/or vaccines, and in developing protein inhibitors as well as discovering new lead compounds.

## Author Contributions

SH, SJ, LR, ST planned the whole work. SH, SJ, LR, ST, and PdC analyzed the data. SH, SJ, LR, ST drafted the manuscript. SH, HF, VA, AS, B, DB, PG, JB, RR, and AT reviewed and analyzed the manuscript. RS, PdC, SJ, and ST performed the literature review and formatting the tables/figures. AU, SK, JB, RR, and JA provided useful comments/suggestions for the improvement of the manuscript.

## Conflict of Interest Statement

The authors declare that the research was conducted in the absence of any commercial or financial relationships that could be construed as a potential conflict of interest.

## Footnotes

1 https://www.ncbi.nlm.nih.gov/genome/genomes/1025?

2 http://www.mholline.lncc.br

3 www.wwpdb.org

4 http://web.expasy.org/protparam/

## Supplementary Material

FIGURE S1 Protein–protein interactome for the identified common conserved targets. Ribosomal pathway protein (VN94_02915 rplJ, rpsH) was interacting with each other either directly or indirectly. The lines color indicates evidences of interactions that were predicted or experimentally validated.

TABLE S1 Tabulated are the 401 conserved target proteins with appropraite 3D structures that were used for further druggability etc. analyse.

TABLE S2 Summary of core Global Druggable (GD) targets along the 13 strains of C. diphtheriae with predicted druggability scores (137 GD targets).
