# Supplementary figures and images for: The Druggable Pocketome of Corynebacterium diphtheriae: A New Approach for in silico Putative Druggable Targets
Source: Front Genet. 2018 Feb 13;9:44. doi: 10.3389/fgene.2018.00044 (PMC5816920; doi:10.3389/fgene.2018.00044)

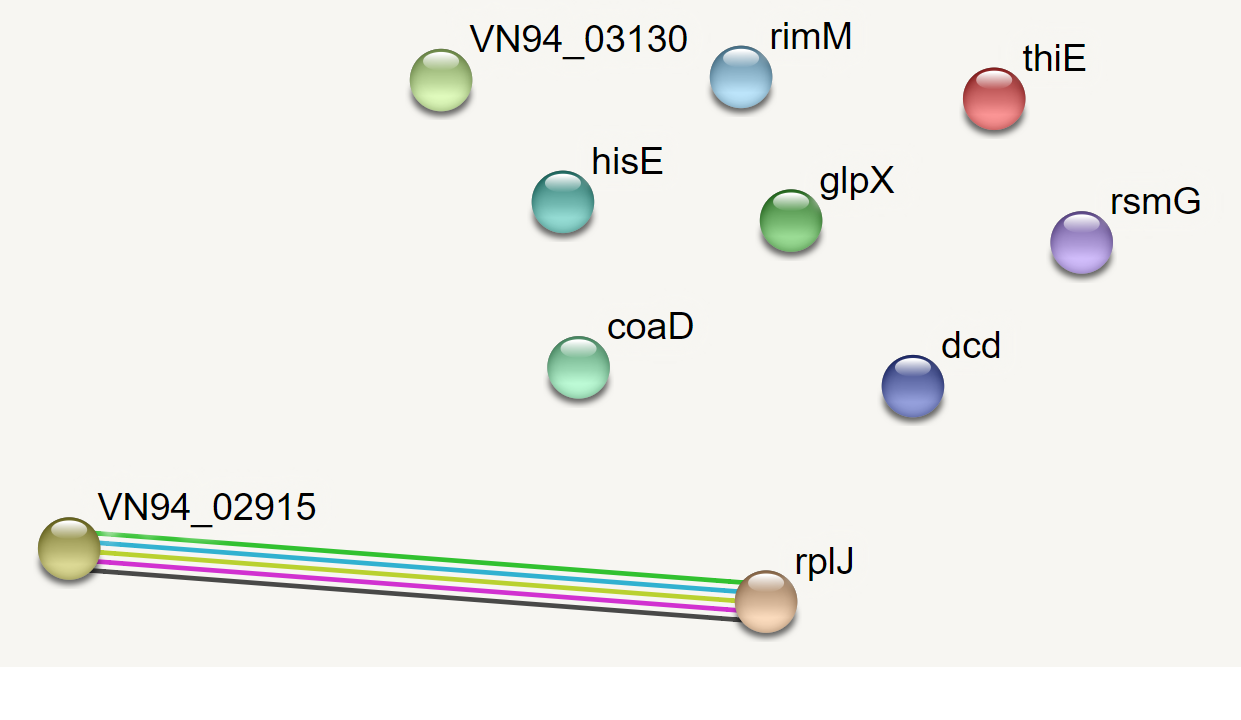

Supplement: FIGURE S1 — Protein–protein interactome for the identified common conserved targets. Ribosomal pathway protein (VN94_02915 rplJ, rpsH) was interacting with each other either directly or indirectly. The lines color indicates evidences of interactions that were predicted or experimentally validated. [file Image_1.tif]
