# Supplementary material for: The Druggable Pocketome of Corynebacterium diphtheriae: A New Approach for in silico Putative Druggable Targets
Source: Front Genet. 2018 Feb 13;9:44. doi: 10.3389/fgene.2018.00044 (PMC5816920; doi:10.3389/fgene.2018.00044)
Supplement: TABLE S1 — Tabulated are the 401 conserved target proteins with appropraite 3D structures that were used for further druggability etc. analyse. [file Table_1.DOC]

**Supplementary table 1:** Tabulated are the 401 conserved target proteins with appropraite 3D structures that were used for further druggability etc. analyses.

| **S.No** | **ID** | **Names** |
| --- | --- | --- |
|  | 38233957 | preprotein translocase subunit SecD |
|  | 38234346 | nicotinic acid mononucleotide adenylyltransferase |
|  | 38233564 | GTP-binding elongation factor |
|  | 38233262 | ribokinase |
|  | 38234258 | pyruvate dehydrogenase subunit E1 |
|  | 38234854 | leucyl-tRNA synthetase |
|  | 38232987 | succinate dehydrogenase flavoprotein subunit |
|  | 38233683 | aspartyl/glutamyl-tRNA amidotransferase subunit B |
|  | 38233856 | arginine/ornithine transport system ATPase |
|  | 38234182 | cell division protein MraZ |
|  | 38234154 | threonine dehydratase |
|  | 38234679 | phosphoribosylformylglycinamidine synthase |
|  | 38233635 | peptide chain release factor 1 |
|  | 38233918 | carbamoyl phosphate synthase small subunit |
|  | 38233685 | lysine exporter protein |
|  | 38234357 | valyl-tRNA synthetase |
|  | 38233617 | sodium/proline symporter |
|  | 38233150 | adenylate kinase |
|  | 38234442 | 50S ribosomal protein L36 |
|  | 38233037 | 2-succinyl-5-enolpyruvyl-6-hydroxy-3-cyclohexene-1-carboxylate synthase |
|  | 38234270 | DNA primase |
|  | 38234771 | glycerol kinase |
|  | 38233664 | electron transfer flavoprotein subunit beta |
|  | 38233119 | molybdenum cofactor biosynthesis protein A |
|  | 38233757 | N-acetyl-gamma-glutamyl-phosphate reductase |
|  | 38233717 | isopropylmalate isomerase large subunit |
|  | 38233079 | 30S ribosomal protein S12 |
|  | 38233088 | 50S ribosomal protein L2 |
|  | 38232667 | peptidyl-prolyl cis-trans isomerase A |
|  | 38233422 | thymidylate synthase |
|  | 38234421 | ribonuclease PH |
|  | 38234797 | manganese superoxide dismutase |
|  | 38233534 | fumarate hydratase |
|  | 38233682 | 6-phosphofructokinase |
|  | 38233704 | isochorismate synthase |
|  | 38234011 | LexA repressor |
|  | 38233419 | precorrin 6A synthase |
|  | 38233271 | phosphoribosylaminoimidazole carboxylase catalytic subunit |
|  | 38233156 | 30S ribosomal protein S11 |
|  | 38234301 | isopentenyl-diphosphate delta-isomerase |
|  | 38233449 | 50S ribosomal protein L31 |
|  | 38233473 | 4-diphosphocytidyl-2-C-methyl-D-erythritol kinase |
|  | 38233935 | Holliday junction resolvase-like protein |
|  | 38234122 | glutamate dehydrogenase |
|  | 38233510 | L-lysine transport protein |
|  | 38233698 | D-3-phosphoglycerate dehydrogenase |
|  | 38234087 | 30S ribosomal protein S2 |
|  | 38233128 | N-acetylmannosamine-6-phosphate 2-epimerase |
|  | 38234906 | thioredoxin reductase |
|  | 38232898 | aspartate kinase |
|  | 38233786 | cytidylate kinase |
|  | 38232645 | recombination protein F |
|  | 38234646 | orotate phosphoribosyltransferase |
|  | 38233929 | 3-dehydroquinate synthase |
|  | 38233094 | 30S ribosomal protein S17 |
|  | 38233299 | S-adenosyl-L-homocysteine hydrolase |
|  | 38233928 | 3-dehydroquinate dehydratase |
|  | 38234170 | cell division protein FtsZ |
|  | 38233890 | glucose-6-phosphate 1-dehydrogenase |
|  | 38234354 | nucleoside diphosphate kinase |
|  | 38233261 | Maf-like protein |
|  | 38233763 | argininosuccinate synthase |
|  | 38233023 | glutamate-1-semialdehyde aminotransferase |
|  | 38233676 | major facilitator family transmembrane efflux protein |
|  | 38233695 | cation-efflux system integral membrane protein |
|  | 38234136 | imidazole glycerol phosphate synthase subunit HisH |
|  | 38234352 | 50S ribosomal protein L21 |
|  | 38232894 | deoxyribose-phosphate aldolase |
|  | 38233015 | porphobilinogen deaminase |
|  | 38233448 | 50S ribosomal protein L28 |
|  | 38233752 | 50S ribosomal protein L20 |
|  | 38233982 | 1-deoxy-D-xylulose-5-phosphate synthase |
|  | 38234547 | ATP-dependent Clp protease ATP-binding subunit |
|  | 38234214 | dihydrolipoamide acetyltransferase |
|  | 38234210 | cobalamin synthase |
|  | 38234181 | S-adenosyl-methyltransferase MraW |
|  | 38233889 | transaldolase |
|  | 38233738 | 30S ribosomal protein S1 |
|  | 38233047 | 50S ribosomal protein L1 |
|  | 38233050 | 50S ribosomal protein L10 |
|  | 38233021 | uroporphyrinogen decarboxylase |
|  | 38234031 | recombinase A |
|  | 38233159 | 50S ribosomal protein L17 |
|  | 38232882 | DNA polymerase III subunits gamma and tau |
|  | 38234118 | formamidopyrimidine-DNA glycosylase |
|  | 38234838 | DNA protection during starvation protein |
|  | 38233842 | aspartate ammonia-lyase |
|  | 38234141 | histidinol dehydrogenase |
|  | 38233022 | protoporphyrinogen oxidase |
|  | 38233036 | O-succinylbenzoate synthase |
|  | 38233751 | 50S ribosomal protein L35 |
|  | 38234913 | ribonuclease P |
|  | 38232737 | ferrisiderophore receptor Irp6A |
|  | 38233091 | 30S ribosomal protein S3 |
|  | 38234239 | threonine synthase |
|  | 38234057 | ribosome-binding factor A |
|  | 38233750 | translation initiation factor IF-3 |
|  | 38234108 | 16S rRNA-processing protein RimM |
|  | 38233142 | glycosylase |
|  | 38233919 | dihydroorotase |
|  | 38234510 | xanthine/uracil permeases family protein |
|  | 161598672 | prephenate dehydratase |
|  | 38234063 | prolyl-tRNA synthetase |
|  | 38233888 | transketolase |
|  | 38233249 | uracil phosphoribosyltransferase |
|  | 38234020 | GTP-binding protein |
|  | 38234888 | anthranilate phosphoribosyltransferase |
|  | 38233537 | exodeoxyribonuclease VII small subunit |
|  | 38233149 | preprotein translocase subunit SecY |
|  | 161598671 | 30S ribosomal protein S6 |
|  | 38233904 | riboflavin synthase subunit alpha |
|  | 38233157 | 30S ribosomal protein S4 |
|  | 38234164 | isoleucyl-tRNA synthetase |
|  | 38233525 | undecaprenyl pyrophosphate synthetase |
|  | 38233610 | galactokinase |
|  | 38234691 | deoxycytidine triphosphate deaminase |
|  | 38232651 | DNA gyrase subunit A |
|  | 38233662 | NUDIX/MutT family hydrolase |
|  | 38232875 | glutamyl-Q tRNA(Asp) synthetase |
|  | 38234620 | adenylosuccinate synthetase |
|  | 38233087 | 50S ribosomal protein L23 |
|  | 38233629 | arginyl-tRNA synthetase |
|  | 38233705 | glutamyl-tRNA synthetase |
|  | 38234348 | gamma-glutamyl kinase |
|  | 38232738 | membrane protein permease Irp6B |
|  | 38234263 | acyl carrier protein |
|  | 38233521 | transcription elongation factor GreA |
|  | 38233712 | phosphoenolpyruvate carboxylase |
|  | 38233914 | guanylate kinase |
|  | 38233694 | ketol-acid reductoisomerase |
|  | 38232998 | UDP-N-acetylenolpyruvoylglucosamine reductase |
|  | 38233535 | fructose 1,6-bisphosphatase II |
|  | 38233900 | excinuclease ABC subunit C |
|  | 38233926 | elongation factor P |
|  | 38233045 | transcription antitermination protein NusG |
|  | 38233081 | elongation factor G |
|  | 38233051 | 50S ribosomal protein L7/L12 |
|  | 38233807 | NADH dehydrogenase |
|  | 38233527 | pantothenate kinase |
|  | 38233632 | homoserine kinase |
|  | 38233155 | 30S ribosomal protein S13 |
|  | 38233895 | phosphoglycerate kinase |
|  | 38234806 | sugar phosphate antiporter |
|  | 38233892 | 6-phosphogluconolactonase |
|  | 38233939 | aspartyl-tRNA synthetase |
|  | 38234052 | tRNA pseudouridine synthase B |
|  | 38233893 | preprotein translocase subunit SecG |
|  | 38232739 | ATP-binding protein Irp6C |
|  | 38234415 | 4'-phosphopantetheinyl transferase |
|  | 38233330 | membrane transporter |
|  | 38233134 | 50S ribosomal protein L6 |
|  | 38234437 | ribonucleotide-diphosphate reductase subunit alpha |
|  | 38233303 | lipoprotein LpqB |
|  | 38234358 | malate dehydrogenase |
|  | 38233845 | phosphoribosyl-ATP pyrophosphatase |
|  | 38233035 | naphthoate synthase |
|  | 38234364 | trigger factor |
|  | 38233569 | N-succinyldiaminopimelate aminotransferase |
|  | 38234772 | glycerol uptake facilitator protein |
|  | 38233242 | tryptophanyl-tRNA synthetase |
|  | 38232884 | recombination protein RecR |
|  | 38233665 | electron transfer flavoprotein subunit alpha |
|  | 38233948 | thiol peroxidase |
|  | 38233383 | phosphoserine aminotransferase |
|  | 38233138 | 50S ribosomal protein L15 |
|  | 38233937 | recombination factor protein RarA |
|  | 38232671 | thiamine biosynthesis protein ThiC |
|  | 38233281 | Ni/Fe-hydrogenase B-type cytochrome subunit |
|  | 38234487 | amidophosphoribosyltransferase |
|  | 38233544 | GTP-dependent nucleic acid-binding protein EngD |
|  | 38233046 | 50S ribosomal protein L11 |
|  | 38233742 | dephospho-CoA kinase |
|  | 38233973 | threonyl-tRNA synthetase |
|  | 38234293 | coproporphyrinogen III oxidase |
|  | 38232902 | catalase |
|  | 38233759 | acetylglutamate kinase |
|  | 161598678 | ubiquinone/menaquinone biosynthesis methyltransferase |
|  | 38232892 | purine nucleoside phosphorylase |
|  | 38234242 | heme oxygenase |
|  | 38233352 | peptide chain release factor 2 |
|  | 161598676 | glucose-1-phosphate adenylyltransferase |
|  | 38233499 | ribose-phosphate pyrophosphokinase |
|  | 38233170 | 50S ribosomal protein L13 |
|  | 38233084 | 30S ribosomal protein S10 |
|  | 38233771 | inorganic polyphosphate/ATP-NAD kinase |
|  | 38233908 | methionyl-tRNA formyltransferase |
|  | 38233089 | 30S ribosomal protein S19 |
|  | 38233137 | 50S ribosomal protein L30 |
|  | 38232984 | dihydrolipoamide dehydrogenase |
|  | 38234163 | DNA polymerase IV |
|  | 38234823 | replicative DNA helicase |
|  | 38233082 | elongation factor Tu |
|  | 38233630 | diaminopimelate decarboxylase |
|  | 38234000 | UDP-glucose 4-epimerase |
|  | 38234901 | tRNA nucleotidyltransferase |
|  | 38233090 | 50S ribosomal protein L22 |
|  | 38233238 | isocitrate dehydrogenase |
|  | 38233061 | DNA-directed RNA polymerase subunit beta' |
|  | 38233822 | cobalt-precorrin-6x reductase |
|  | 38233962 | Holliday junction resolvase |
|  | 38234051 | bifunctional riboflavin kinase/FMN adenylyltransferase |
|  | 38234030 | recombination regulator RecX |
|  | 38234517 | pyruvate dehydrogenase |
|  | 38234804 | two component system histidine kinase |
|  | 38233447 | 50S ribosomal protein L33 |
|  | 38233507 | transcription-repair coupling factor |
|  | 38234744 | UDP-galactopyranose mutase |
|  | 38233154 | translation initiation factor IF-1 |
|  | 38234079 | 1-deoxy-D-xylulose 5-phosphate reductoisomerase |
|  | 38233313 | 3-phosphoshikimate 1-carboxyvinyltransferase |
|  | 38233497 | 50S ribosomal protein L25 |
|  | 38234428 | nicotinate phosphoribosyltransferase |
|  | 38234212 | leucyl aminopeptidase |
|  | 38232868 | prephenate dehydrogenase |
|  | 38234360 | ATP-dependent protease ATP-binding subunit ClpX |
|  | 38233865 | ferrochelatase |
|  | 38234139 | imidazoleglycerol-phosphate dehydratase |
|  | 38234541 | DNA repair protein RadA |
|  | 38233913 | DNA-directed RNA polymerase subunit omega |
|  | 38233446 | 30S ribosomal protein S14 |
|  | 38234347 | gamma-glutamyl phosphate reductase |
|  | 38234776 | seryl-tRNA synthetase |
|  | 38234824 | 50S ribosomal protein L9 |
|  | 38234074 | cobyric acid synthase |
|  | 38233852 | dihydroorotate dehydrogenase 2 |
|  | 38233736 | DNA polymerase I |
|  | 38233729 | phosphopantetheine adenylyltransferase |
|  | 38233429 | glucose-6-phosphate isomerase |
|  | 38234173 | undecaprenyldiphospho-muramoylpentapeptide beta-N-acetylglucosaminyltransferase |
|  | 38233472 | dimethyladenosine transferase |
|  | 38233168 | ESAT-6-like protein |
|  | 38234199 | ubiquinol-cytochrome C reductase cytochrome B subunit |
|  | 38234202 | cytochrome C oxidase subunit III |
|  | 38233673 | aspartyl/glutamyl-tRNA amidotransferase subunit C |
|  | 38234201 | ubiquinol-cytochrome C reductase cytochrome C subunit |
|  | 38233017 | delta-aminolevulinic acid dehydratase |
|  | 161598673 | cobyrinic acid a,c-diamide synthase |
|  | 38233135 | 50S ribosomal protein L18 |
|  | 38233906 | ribulose-phosphate 3-epimerase |
|  | 38233477 | RNA polymerase ECF-subfamily sigma factor |
|  | 38233954 | adenine phosphoribosyltransferase |
|  | 38234211 | branched-chain amino acid aminotransferase |
|  | 38233539 | 4-hydroxy-3-methylbut-2-enyl diphosphate reductase |
|  | 38234492 | glutathione peroxidase |
|  | 38233513 | phosphopyruvate hydratase |
|  | 38233538 | exodeoxyribonuclease VII large subunit |
|  | 38233631 | homoserine dehydrogenase |
|  | 38233787 | GTP-binding protein EngA |
|  | 38234140 | histidinol-phosphate aminotransferase |
|  | 38233931 | chorismate synthase |
|  | 38234086 | elongation factor Ts |
|  | 38234175 | UDP-N-acetylmuramoyl-L-alanyl-D-glutamate synthetase |
|  | 38233093 | 50S ribosomal protein L29 |
|  | 38233999 | diphtheria toxin repressor |
|  | 38233275 | hydrogenase expression/formation protein HypE |
|  | 38233744 | excinuclease ABC subunit B |
|  | 38233690 | dihydroxy-acid dehydratase |
|  | 38234605 | cardiolipin synthase |
|  | 38233230 | homoserine O-acetyltransferase |
|  | 38234216 | lipoyl synthase |
|  | 38233674 | aspartyl/glutamyl-tRNA amidotransferase subunit A |
|  | 38234643 | fructose-bisphosphate aldolase |
|  | 38234060 | transcription elongation factor NusA |
|  | 38233086 | 50S ribosomal protein L4 |
|  | 38233850 | undecaprenyl pyrophosphate phosphatase |
|  | 38233092 | 50S ribosomal protein L16 |
|  | 38234723 | phosphoenolpyruvate carboxykinase |
|  | 38234129 | prolipoprotein diacylglyceryl transferase |
|  | 38234337 | 30S ribosomal protein S20 |
|  | 161598680 | DNA polymerase III subunit beta |
|  | 38233445 | 30S ribosomal protein S18 |
|  | 38234191 | phospho-2-dehydro-3-deoxyheptonate aldolase |
|  | 38234104 | 50S ribosomal protein L19 |
|  | 38233252 | flavoprotein disulfide reductase |
|  | 38234423 | glutamate racemase |
|  | 38234324 | inosine-uridine preferring nucleoside hydrolase |
|  | 38234461 | serine acetyltransferase |
|  | 38234567 | hypoxanthine-guanine phosphoribosyltransferase |
|  | 38232925 | endonuclease III |
|  | 38233989 | polyphosphate glucokinase |
|  | 38234328 | Mrr restriction system protein |
|  | 38232647 | DNA gyrase subunit B |
|  | 38233044 | preprotein translocase subunit SecE |
|  | 38234110 | signal recognition particle protein |
|  | 38233760 | acetylornithine aminotransferase |
|  | 38233699 | 3-isopropylmalate dehydrogenase |
|  | 38234176 | phospho-N-acetylmuramoyl-pentapeptide-transferase |
|  | 38234611 | acetate kinase |
|  | 38233902 | 6,7-dimethyl-8-ribityllumazine synthase |
|  | 38234427 | ATP-dependent Clp protease adaptor protein ClpS |
|  | 38234742 | high-affinity choline transport protein |
|  | 38233678 | iron ABC transporter membrane protein |
|  | 38233930 | shikimate kinase |
|  | 38234162 | L-asparaginase |
|  | 38234015 | phosphofructokinase |
|  | 38233136 | 30S ribosomal protein S5 |
|  | 38232672 | thiamine-phosphate pyrophosphorylase |
|  | 38233887 | protoheme IX farnesyltransferase |
|  | 38234286 | pyridoxamine kinase |
|  | 38233912 | bifunctional phosphopantothenoylcysteine decarboxylase/phosphopantothenate synthase |
|  | 38233936 | alanyl-tRNA synthetase |
|  | 38234443 | NAD synthetase |
|  | 38234452 | phosphoglucomutase |
|  | 161598674 | aconitate hydratase |
|  | 38233609 | galactose-1-phosphate uridylyltransferase |
|  | 38233805 | magnesium/cobalt transporter CorA |
|  | 38234887 | anthranilate synthase component II |
|  | 38232675 | thiazole synthase |
|  | 38233844 | ATP phosphoribosyltransferase |
|  | 38233766 | tyrosyl-tRNA synthetase |
|  | 38233202 | GMP synthase |
|  | 38233820 | precorrin-8X methylmutase |
|  | 38233184 | co-chaperonin GroES |
|  | 38234200 | ubiquinol-cytochrome C reductase iron-sulfur protein |
|  | 38233456 | large-conductance mechanosensitive channel |
|  | 38234236 | MFS family transporter |
|  | 38233817 | cobaltochelatase subunit CobN |
|  | 38234413 | bacterioferritin comigratory protein |
|  | 38233273 | hydrogenase isoenzyme formation protein |
|  | 38233158 | DNA-directed RNA polymerase subunit alpha |
|  | 38234085 | uridylate kinase |
|  | 38234009 | transcriptional regulator NrdR |
|  | 38233755 | phenylalanyl-tRNA synthetase subunit alpha |
|  | 38234111 | PII uridylyl-transferase |
|  | 38233894 | triosephosphate isomerase |
|  | 38234109 | 30S ribosomal protein S16 |
|  | 38233085 | 50S ribosomal protein L3 |
|  | 38232953 | adenylate cyclase |
|  | 38234669 | molecular chaperone DnaK |
|  | 38233375 | cold shock protein B |
|  | 38234023 | diaminopimelate epimerase |
|  | 38234049 | 30S ribosomal protein S15 |
|  | 38233917 | carbamoyl phosphate synthase large subunit |
|  | 38234071 | malate:quinone oxidoreductase |
|  | 38234132 | phosphoribosyl-AMP cyclohydrolase |
|  | 38234351 | 50S ribosomal protein L27 |
|  | 38233761 | ornithine carbamoyltransferase |
|  | 38234047 | dihydrodipicolinate reductase |
|  | 38234483 | 4-amino-4-deoxychorismate lyase |
|  | 38233171 | 30S ribosomal protein S9 |
|  | 38234077 | 4-hydroxy-3-methylbut-2-en-1-yl diphosphate synthase |
|  | 38233764 | argininosuccinate lyase |
|  | 38233970 | lipid A biosynthesis lauroyl acyltransferase |
|  | 38234194 | glucose kinase |
|  | 38233692 | acetolactate synthase 1 catalytic subunit |
|  | 38233824 | precorrin-6Y C5,15-methyltransferase |
|  | 38233568 | ferredoxin |
|  | 38233253 | pyruvate carboxylase |
|  | 38233803 | 6-phosphogluconate dehydrogenase |
|  | 38233916 | orotidine 5'-phosphate decarboxylase |
|  | 38234894 | tryptophan synthase subunit alpha |
|  | 38234460 | cysteine synthase |
|  | 38233354 | cell division ATP-binding protein |
|  | 38234128 | pyruvate kinase |
|  | 161598677 | type II citrate synthase |
|  | 38233756 | phenylalanyl-tRNA synthetase subunit beta |
|  | 38234094 | ribonuclease HII |
|  | 38233177 | alanine racemase |
|  | 38233724 | uracil-DNA glycosylase |
|  | 38233080 | 30S ribosomal protein S7 |
|  | 38233720 | NAD(P)H-dependent glycerol-3-phosphate dehydrogenase |
|  | 38234911 | 16S rRNA methyltransferase GidB |
|  | 38233857 | methylmalonyl-CoA mutase |
|  | 38232900 | aspartate-semialdehyde dehydrogenase |
|  | 38234084 | ribosome recycling factor |
|  | 38233274 | hydrogenase expression/formation protein HypC |
|  | 38234537 | 2-C-methyl-D-erythritol 4-phosphate cytidylyltransferase |
|  | 38233528 | serine hydroxymethyltransferase |
|  | 38233133 | 30S ribosomal protein S8 |
|  | 38234495 | adenylosuccinate lyase |
|  | 38233946 | histidyl-tRNA synthetase |
|  | 38233718 | isopropylmalate isomerase small subunit |
|  | 38234457 | UDP-N-acetylglucosamine 1-carboxyvinyltransferase |
|  | 38233634 | transcription termination factor Rho |
|  | 38233956 | preprotein translocase subunit SecF |
|  | 38234119 | ribonuclease III |
|  | 38233450 | 50S ribosomal protein L32 |
|  | 38233723 | thiamine monophosphate kinase |
|  | 38234279 | glycyl-tRNA synthetase |
|  | 38232948 | DNA topoisomerase I |
|  | 38233234 | Iron-related transport system membrane protein |
|  | 38233469 | methionyl-tRNA synthetase |
|  | 38234133 | imidazole glycerol phosphate synthase subunit HisF |
|  | 38232876 | gluconate permease |
|  | 38233546 | 2-dehydropantoate 2-reductase |
|  | 38233577 | succinyl-diaminopimelate desuccinylase |
|  | 38234472 | succinyl-CoA:coenzyme A transferase |
|  | 38232976 | glucose-1-phosphate thymidylyltransferase |
|  | 38233300 | thymidylate kinase |
|  | 38233014 | glutamyl-tRNA reductase |
|  | 38234384 | oligoribonuclease |
|  | 161598679 | 2-isopropylmalate synthase |
|  | 38233270 | phosphoribosylaminoimidazole carboxylase ATPase subunit |
|  | 38234914 | 50S ribosomal protein L34 |
|  | 38234743 | choline dehydrogenase |
|  | 38233160 | tRNA pseudouridine synthase A |
|  | 38233721 | D-alanyl-alanine synthetase A |
|  | 38234073 | mycothione reductase |
|  | 38233077 | anaerobic ribonucleoside triphosphate reductase |
|  | 38233836 | ATPase |
|  | 38234737 | phosphoribose diphosphate:decaprenyl-phosphate phosphoribosyltransferase |

**Supplementary table 2: Summary of core Global Druggable (GD) targets along the 13 strains of *C. diphtheriae* with predicted druggability scores**

(137 GD targets).

| **S. No** | **Protein Loci** | **Names of GD Protein Targets** | **Drugability Scores** | **S. No** | **Protein Loci** | **Names of GD Protein Targets** | **Drugability Scores** |
| --- | --- | --- | --- | --- | --- | --- | --- |
| 1 | 38233090 | 50S ribosomal protein L22 | 0.986 | 70 | 38233676 | Major facilitator family transmembrane efflux protein | 0.8232 |
| 2 | 38233159 | 50S ribosomal protein L17 | 0.9721 | 71 | 38234472 | Succinyl-CoA:coenzyme A transferase | 0.8225 |
| 3 | 38234415 | 4'-phosphopantetheinyl transferase | 0.9679 | 72 | 1.62E+08 | Ubiquinone/menaquinone biosynthesis methyltransferase | 0.8221 |
| 4 | 38233170 | 50S ribosomal protein L13 | 0.9636 | 73 | 38233824 | Precorrin-6Y C5, 15-methyltransferase | 0.8186 |
| 5 | 38234015 | Phosphofructokinase | 0.9579 | 74 | 38234293 | Coproporphyrinogen III oxidase | 0.8173 |
| 6 | 38233632 | Homoserine kinase | 0.9514 | 75 | 38233902 | 6,7-dimethyl-8-ribityllumazine synthase | 0.8169 |
| 7 | 38234347 | Gamma-glutamyl phosphate reductase | 0.9477 | 76 | 38233046 | 50S ribosomal protein L11 | 0.8146 |
| 8 | 38233630 | Diaminopimelate decarboxylase | 0.9468 | 77 | 38233865 | Ferrochelatase | 0.8089 |
| 9 | 38233771 | Inorganic polyphosphate/ATP-NAD kinase | 0.9428 | 78 | 38234646 | Orotate phosphoribosyltransferase | 0.8086 |
| 10 | 38233820 | Precorrin-8X methylmutase | 0.9428 | 79 | 38234163 | DNA polymerase IV | 0.8048 |
| 11 | 38233014 | Glutamyl-tRNA reductase | 0.9405 | 80 | 38233889 | Transaldolase | 0.8039 |
| 12 | 38234202 | Cytochrome C oxidase subunit III | 0.9385 | 81 | 38233177 | Alanine racemase | 0.7999 |
| 13 | 38234413 | Bacterioferritin comigratory protein | 0.9382 | 82 | 38233274 | Hydrogenase expression/formation protein HypC | 0.7992 |
| 14 | 38233080 | 30S ribosomal protein S7 | 0.9355 | 83 | 38234888 | Anthranilate phosphoribosyltransferase | 0.7951 |
| 15 | 38234324 | Inosine-uridine preferring nucleoside hydrolase | 0.9332 | 84 | 38233936 | Alanyl-tRNA synthetase | 0.7938 |
| 16 | 38233422 | Thymidylate synthase | 0.9328 | 85 | 38233704 | Isochorismate synthase | 0.7917 |
| 17 | 38233962 | Holliday junction resolvase | 0.9285 | 86 | 38234182 | Cell division protein MraZ | 0.7864 |
| 18 | 38233720 | NADPH-dependent glycerol-3-phosphate dehydrogenase | 0.9269 | 87 | 38233275 | Hydrogenase expression/formation protein HypE | 0.7863 |
| 19 | 38233894 | Triosephosphate isomerase | 0.92 | 88 | 38232868 | Prephenate dehydrogenase | 0.782 |
| 20 | 1.62E+08 | 30S ribosomal protein S6 | 0.9186 | 89 | 38233234 | Iron-related transport system membrane protein | 0.782 |
| 21 | 38233281 | Ni/Fe-hydrogenase B-type cytochrome subunit | 0.9126 | 90 | 38234771 | Glycerol kinase | 0.7805 |
| 22 | 38233913 | DNA-directed RNA polymerase subunit omega | 0.9118 | 91 | 38234108 | 16S rRNA-processing protein RimM | 0.7778 |
| 23 | 38233673 | Aspartyl/glutamyl-tRNA amidotransferase subunit C | 0.9103 | 92 | 38234110 | Signal recognition particle protein | 0.777 |
| 24 | 38234510 | Xanthine/uracil permeases family protein | 0.9102 | 93 | 38234162 | L-asparaginase | 0.7735 |
| 25 | 38233133 | 30S ribosomal protein S8 | 0.907 | 94 | 38233926 | Elongation factor P | 0.7732 |
| 26 | 38233662 | NUDIX/MutT family hydrolase | 0.906 | 95 | 38234691 | Deoxycytidine triphosphate deaminase | 0.7728 |
| 27 | 38234742 | High-affinity choline transport protein | 0.9057 | 96 | 38233937 | Recombination factor protein RarA | 0.7723 |
| 28 | 38233082 | Elongation factor Tu | 0.9043 | 97 | 38233050 | 50S ribosomal protein L10 | 0.7684 |
| 29 | 38234242 | Heme oxygenase | 0.9004 | 98 | 38234772 | Glycerol uptake facilitator protein | 0.7677 |
| 30 | 38232898 | Aspartate kinase | 0.8972 | 99 | 38233330 | Membrane transporter | 0.7672 |
| 31 | 38233249 | Uracil phosphoribosyltransferase | 0.8879 | 100 | 38233928 | 3-dehydroquinate dehydratase | 0.7631 |
| 32 | 38234279 | Glycyl-tRNA synthetase | 0.8868 | 101 | 38233061 | DNA-directed RNA polymerase subunit beta' | 0.7626 |
| 33 | 38234887 | Anthranilate synthase component II | 0.8867 | 102 | 38233546 | 2-dehydropantoate 2-reductase | 0.761 |
| 34 | 38233134 | 50S ribosomal protein L6 | 0.8857 | 103 | 38233270 | Phosphoribosylaminoimidazole carboxylase ATPase subunit | 0.7608 |
| 35 | 38233079 | 30S ribosomal protein S12 | 0.8854 | 104 | 38233699 | 3-isopropylmalate dehydrogenase | 0.7607 |
| 36 | 38233051 | 50S ribosomal protein L7/L12 | 0.8849 | 105 | 38234457 | UDP-N-acetylglucosamine 1-carboxyvinyltransferase | 0.757 |
| 37 | 38233617 | Sodium/proline symporter | 0.8842 | 106 | 38233521 | Transcription elongation factor GreA | 0.7551 |
| 38 | 38233156 | 30S ribosomal protein S11 | 0.882 | 107 | 38233037 | 2-succinyl-5-enolpyruvyl-6-hydroxy-3-cyclohexene-1-carboxylate synthase | 0.7541 |
| 39 | 38233534 | Fumarate hydratase | 0.8771 | 108 | 38234824 | 50S ribosomal protein L9 | 0.7529 |
| 40 | 38233525 | Undecaprenyl pyrophosphate synthetase | 0.8767 | 109 | 38234270 | DNA primase | 0.7525 |
| 41 | 38234199 | Ubiquinol-cytochrome C reductase cytochrome B subunit | 0.8753 | 110 | 38234911 | 16S rRNA methyltransferase GidB | 0.7521 |
| 42 | 38234346 | Nicotinic acid mononucleotide adenylyltransferase | 0.8684 | 111 | 38232902 | Catalase | 0.7515 |
| 43 | 38234109 | 30S ribosomal protein S16 | 0.8679 | 112 | 38233035 | Naphthoate synthase | 0.7514 |
| 44 | 38234118 | Formamidopyrimidine-DNA glycosylase | 0.8635 | 113 | 38233313 | 3-phosphoshikimate 1-carboxyvinyltransferase | 0.7507 |
| 45 | 38234804 | Two component system histidine kinase | 0.863 | 114 | 38233683 | aspartyl/glutamyl-tRNA amidotransferase subunit B | 0.7504 |
| 46 | 38233092 | 50S ribosomal protein L16 | 0.8597 | 115 | 38233300 | Thymidylate kinase | 0.7492 |
| 47 | 38233757 | N-acetyl-gamma-glutamyl-phosphate reductase | 0.8588 | 116 | 38233086 | 50S ribosomal protein L4 | 0.7483 |
| 48 | 38234806 | Sugar phosphate antiporter | 0.8578 | 117 | 1.62E+08 | Prephenate dehydratase | 0.7478 |
| 49 | 38233154 | Translation initiation factor IF-1 | 0.8498 | 118 | 38233017 | Delta-aminolevulinic acid dehydratase | 0.7473 |
| 50 | 38233717 | Isopropylmalate isomerase large subunit | 0.846 | 119 | 38234176 | Phospho-N-acetylmuramoyl-pentapeptide-transferase | 0.746 |
| 51 | 38234492 | Glutathione peroxidase | 0.8455 | 120 | 38233755 | Phenylalanyl-tRNA synthetase subunit alpha | 0.7458 |
| 52 | 38233729 | Phosphopantetheine adenylyltransferase | 0.8449 | 121 | 38233419 | Precorrin 6A synthase | 0.7454 |
| 53 | 38233845 | Phosphoribosyl-ATP pyrophosphatase | 0.8416 | 122 | 38233273 | Hydrogenase isoenzyme formation protein | 0.7432 |
| 54 | 38234020 | GTP-binding protein | 0.8383 | 123 | 38233021 | Uroporphyrinogen decarboxylase | 0.7418 |
| 55 | 38234384 | Oligoribonuclease | 0.8383 | 124 | 38233918 | Carbamoyl phosphate synthase small subunit | 0.7397 |
| 56 | 38233721 | D-alanyl-alanine synthetase A | 0.8378 | 125 | 38234057 | Ribosome-binding factor A | 0.7365 |
| 57 | 38233609 | Galactose-1-phosphate uridylyltransferase | 0.835 | 126 | 38233836 | ATPase | 0.7361 |
| 58 | 38233664 | Electron transfer flavoprotein subunit beta | 0.8338 | 127 | 38233935 | Holliday junction resolvase-like protein | 0.7302 |
| 59 | 38233202 | GMP synthase | 0.8311 | 128 | 1.62E+08 | 2-isopropylmalate synthase | 0.7298 |
| 60 | 38234214 | Dihydrolipoamide acetyltransferase | 0.8298 | 129 | 38232672 | Thiamine-phosphate pyrophosphorylase | 0.7231 |
| 61 | 38233535 | Fructose 1,6-bisphosphatase II | 0.828 | 130 | 38234906 | Thioredoxin reductase | 0.7217 |
| 62 | 1.62E+08 | DNA polymerase III subunit beta | 0.8272 | 131 | 38233807 | NADH dehydrogenase | 0.7129 |
| 63 | 38233242 | Tryptophanyl-tRNA synthetase | 0.8269 | 132 | 38232948 | DNA topoisomerase I | 0.7114 |
| 64 | 38233766 | Tyrosyl-tRNA synthetase | 0.8269 | 133 | 38233477 | RNA polymerase ECF-subfamily sigma factor | 0.7114 |
| 65 | 38234011 | LexA repressor | 0.8263 | 134 | 38234901 | tRNA nucleotidyltransferase | 0.7073 |
| 66 | 38234136 | Imidazole glycerol phosphate synthase subunit HisH | 0.8252 | 135 | 38233744 | Excinuclease ABC subunit B | 0.7045 |
| 67 | 38234483 | 4-amino-4-deoxychorismate lyase | 0.8249 | 136 | 38233906 | Ribulose-phosphate 3-epimerase | 0.7036 |
| 68 | 38234051 | Bifunctional riboflavin kinase/FMN adenylyltransferase | 0.824 | 137 | 38233047 | 50S ribosomal protein L1 | 0.7012 |
| 69 | 38234119 | Ribonuclease III | 0.8236 |  | | | |
